# UITOTO: a software for generating molecular diagnoses for species descriptions

**DOI:** 10.1111/cla.70023

**Published:** 2025-12-20

**Authors:** Ambrosio Torres, Leshon Lee, Amrita Srivathsan, Rudolf Meier

**Affiliations:** ^1^ Center for Integrative Biodiversity Discovery Leibniz Institute for Evolution and Biodiversity Science Berlin Germany; ^2^ Lee Kong Chian Natural History Museum National University of Singapore Singapore Singapore

## Abstract

Millions of species remain undescribed, and each eventually will require a species description with a diagnosis. Yet, we lack software that can derive state‐specific and contrastive molecular diagnoses and allows the user to validate them based on all available sequences for the taxon under study. Here we introduce UITOTO, which addresses this shortcoming by facilitating the identification, testing, and visualization of diagnostic molecular combinations (DMCs). The software uses a weighted random sampling algorithm based on the Jaccard Index for building candidate DMCs. It then selects DMCs with the highest specificity stability, meeting user‐defined thresholds for exclusive character states. If multiple optimal DMCs are identified, UITOTO derives a majority‐consensus DMC. To verify whether the generated DMCs are contrastive, UITOTO includes a validation module that tests DMCs against databases, efficiently handling thousands of aligned or unaligned sequences. We here, not only propose UITOTO, but also assess its performance relative to other software that can derive DMCs (e.g. MOLD). For this purpose, we analyse three large empirical datasets: (i) *Megaselia* (Diptera: Phoridae: 69 species, 2229 training and 30 289 testing barcodes); (ii) Mycetophilidae (Diptera: 118 species, 1456 training, 60 349 testing barcodes); and (iii) European Lepidoptera (49 species, 591 training, 21 483 testing barcodes). Based on classification metrics (e.g. F1 Score), UITOTO's DMCs outcompete DMCs from other software. We furthermore provide guidelines for generating molecular diagnoses and a user‐friendly Shiny App‐GUI that includes a module for obtaining publication‐quality DMC visualizations. Overall, our study confirms that the biggest challenge for generating molecular and morphological diagnoses is similar: balancing specificity and length; short diagnoses often lack specificity, while excessively long DMCs are often so specific that they do not accommodate intraspecific variation.

## Introduction

Humans have long classified biological diversity in order to make sense of the living world (Llorente‐Bousquets, 1990). This effort culminated in a set of rules for naming and classifying both the known and the millions of unknown species. Completing the description of this unknown diversity is now one of the most important unfinished tasks in biology, which is becoming increasingly urgent because biodiversity loss is projected to be among the 10 most pressing global challenges of the coming decade (World Economic Forum, 2025). The assignment of scientific names will be essential for filing biological data and thus meaningful holistic biomonitoring (Meier et al., 2024).

The principles and data used for classifying and describing species have changed substantially over time. These developments include the incorporation of new evidence types such as DNA sequences, gene maps, biochemical profiles, allozymes, ecological attributes and biogeographical patterns into species delimitation (Llorente‐Bousquets, 1990; Renner, 2016; Braby et al., 2024). The resulting expansion of available data has created a more comprehensive toolkit for exploring and interpreting biological diversity (Meier, 2008; Ahrens, 2024; Janzen et al., 2017; Maltsev and Erst, 2023). At the same time, the introduction of these additional data types has required the development of new analytical frameworks for formulating species hypotheses. Numerous methods and software packages have been introduced for analysing DNA sequence data generated from single loci or from multiple loci. These include methods that rely on distance‐ or tree‐based estimation techniques such as ABGD (Puillandre et al., 2012), ASAP (Puillandre et al., 2021), the Poisson tree process (PTP; Zhang et al., 2013) and the general mixed Yule coalescent model (GMYC; Pons et al., 2006), while other approaches employ multispecies coalescent model‐based methods (e.g. BPP; Flouri et al., 2018) or are based on trinomial distributions of triplets (tr2; Fujisawa et al., 2016). Comprehensive reviews of these techniques can be found in Hubert et al. (2024), Miralles et al. (2024) and Meier et al. (2025).

Surprisingly, much less attention has been given to the next step in the taxonomic workflow: producing molecular diagnoses for formal species descriptions. Yet, this step is conceptually distinct from species delimitation. One groups specimens into species, whereas a description with diagnosis summarizes the evidence distinguishing a newly proposed species from all others. Unfortunately, currently many species descriptions include DNA “diagnoses” that do not satisfy nomenclatural codes. To address these shortcomings the International Commission on Zoological Nomenclature (ICZN) recently published recommendations to prevent future problems like the ones caused by Sharkey *et al*.'s description of more than 400 species (2021). These descriptions used consensus barcodes as diagnoses without specifying diagnostic positions in the sequences (Fedosov et al., 2022; Meier et al., 2022; Rheindt et al., 2023). ICZN thus reemphasized the need for DNA diagnoses to be state‐specific (provide distinguishing molecular sites) and contrastive (demonstrate that states are only found in one known species). One way to satisfy these criteria is to provide a diagnostic molecular combination (DMC): a unique group of exclusive molecular character states that identify a particular species relative to a reference sequence that must also be specified (e.g. the sequence of the holotype). For example, the DMC of a species could be expressed as “[25: A; 69: G]”, indicating that the species has an Adenine at position 25 and a Guanine at position 69 (relative to a reference sequence), which can be contrasted against other species using sequence alignments. In practice, DMCs could consist of a single diagnostic site but many species lack such unique sites (see e.g. Marchán et al., 2020), so that DMCs consisting of multiple sites are needed.

The growing recognition that DNA‐based diagnoses will become an integral part of taxonomic practice (Emerson, 2025) has spurred the development of approaches for generating molecular diagnoses across diverse groups. For example, for fungi Tedersoo et al. (2024) developed methods for describing new species using short, fixed‐length barcodes (20–30 bp) from the internal transcribed spacer (ITS) and Large Subunit ribosomal RNA (LSU) regions, selecting continuous fragments with no ambiguity within the target species and requiring at least two nucleotide differences from close relatives. Similarly, Flores‐Olvera et al. (2016) proposed molecular diagnoses for plant species based on state‐specific nucleotide characters across multiple plastid and nuclear loci. Both approaches satisfied different International Codes of Nomenclature for multicellular species (ICNafp: Turland et al., 2018; ICZN: International Commission on Zoological Nomenclature, 1999), but these diagnoses are still derived manually although ideally, diagnoses should be derived by algorithmic procedures to improve repeatability and the ability to analyse very large datasets. Such algorithmic solutions have been proposed for animal species because many can be distinguished based on a single mitochondrial gene. The most popular gene is the protein‐encoding “DNA barcode” gene (COI) that is largely intron‐free and thus comparatively easy to align. This simplifies the process of comparing large numbers of sequences and providing the exact position of diagnostic sites.

Several methods and software packages for deriving DNA diagnoses mostly used for animal species have been proposed (Table 1; Fedosov et al., 2022; Brower and DeSalle, 2024). They include Cladistic Haplotype Aggregation (CHA; Brower, 1999), Spider (Brown et al., 2012); BLOG (Weitschek et al., 2013); FASTACHAR (Merckelbach and Borges, 2020); QUIDDICH (Kühn and Haase, 2020); DeSignate (Hütter et al., 2020), MOLD (Fedosov et al., 2022. New version at: https://itaxotools.org/index.html) and CAOS‐R (Sarkar et al., 2008; Bergmann, 2024). All methods constitute important steps towards obtaining rigorous diagnoses but also exhibit several limitations. First, some tools such as FASTACHAR (Merckelbach and Borges, 2020) search only for single‐site DMCs, which may be absent in some species and can become unreliable when newly sampled haplotypes lack the designated diagnostic state, illustrating the broader problem that overly short DMCs may fail to capture intraspecific variability. Second, packages that allow multi‐site DMCs often provide no rigorous mechanism for verifying whether the proposed DMCs remain diagnostic when compared against all available sequences for a taxon, which is increasingly problematic as barcode datasets grow. Third, most existing software was designed for relatively small datasets with only hundreds of sequences, yet modern barcoding efforts routinely generate thousands of sequences and will do so even more frequently as species descriptions expand to include “dark taxa” (Srivathsan et al., 2019, 2023; Amorim et al., 2025; Caruso et al., 2024; Meier et al., 2024). In addition, many of these tools lack user‐friendly graphical interfaces even though such interfaces are essential for broad taxonomic uptake of molecular diagnoses (see Table 1).

**Table 1:** General comparison of available software tools for molecular diagnosis in taxonomy. The table indicates the available functionalities in each one of the software.

Software	References	DMCs identification	Taxonomic identification	Diagnosis visualization	GUI	Online access	Tree‐based analysis	Source
UITOTO	This study	Yes	Yes	Yes	Yes	Yes	No	https://github.com/atorresgalvis/UITOTO
CAOS /CAOS‐R	Sarkar et al. (2008)/Bergmann (2024)	No*	Yes^†^	No	No	No	Yes	https://github.com/JuliaHealth/CAOS.jl/https://github.com/M0rph3u2x/CAOS‐R
MOLD	Fedosov et al. (2022)	Yes	No	No	Yes	Yes	No	https://github.com/SashaFedosov/MolD
DeSignate	Hütter et al. (2020)	Yes	No	Yes	Yes	Yes	Yes/No	https://github.com/DatabaseGroup/DeSignate
QUIDDICH	Kühn and Haase (2020)	No*	No	No	No	No	No	https://cran.r‐project.org/package=quiddich
FASTACHAR	Merckelbach and Borges (2020)	No*	No	No	Yes	No	No	https://github.com/smerckel/FastaChar
BLOG	Weitschek et al. (2013)	Yes	Yes	No	Yes	No	No	http://dmb.iasi.cnr.it/blog‐downloads.php
Spider	Brown et al. (2012)	No*	No	No	No	No	No	https://cran.r‐project.org/web/packages/spider/

GUI, graphical user interface; DMCs, diagnostic molecular combination.

* Tools specifically designed to identify single nucleotide characters (rather than combinations of characters) for diagnosing species. These tools typically aim to detect apomorphic characters, which are diagnostic sites where all sequences of a query taxon (e.g. a new species) have nucleotide(s) not found in any member of the remaining taxa. However, such diagnostic characters are seldom present for every taxon in the analysed alignments, especially in high‐throughput taxonomy where hundreds of species and thousands of sequences are involved.

^†^ These tools use the identified nucleotide diagnostic characters and apply a hybrid approach combining elements of decision trees and distance methods for taxonomic identification.

Here, we introduce UITOTO, a user‐friendly R package (R Core Team, 2024) specifically developed to tackle the challenges associated with finding, testing, and visualizing reliable diagnostic molecular combinations (DMCs) in large datasets. We demonstrate that UITOTO produces reliable DMCs for most of the species in three different large empirical datasets: (i) European Lepidoptera, with 49 species, 591 barcodes for training, and 21 483 for testing (Dincă et al., 2021); (ii) family Mycetophilidae (Diptera: Bibionomorpha), with 118 species, 1456 barcodes for training (Amorim et al., 2024), and 60 349 for testing (Ratnasingham and Hebert, 2007); and (iii) genus *Megaselia* (Diptera: Phoridae), with DMCs for 69 species (Lee, 2023), comprising 2229 barcodes in the training dataset and 30 289 in the testing dataset. The datasets are realistic in that they include many “difficult” species where balancing DMC length and specificity is particularly difficult. We use these datasets to provide guidelines and instructions for obtaining DMCs with UITOTO.

## Materials and Methods

### Description of the UITOTO R package

UITOTO (User InTerface for Optimal molecular diagnoses in high‐throughput TaxOnomy) is an R package (R Core Team, 2024) suitable for all major operating systems that facilitates the discovery, testing, and visualization of optimal Diagnostic Molecular Combinations (DMCs) from datasets with thousands of sequences. The name UITOTO is a tribute to the indigenous Uitoto (also Huitoto or Witoto) people of the Colombian‐Peruvian Amazon, whose deep understanding of biodiversity and commitment to preserving their natural environment in the face of the challenges they face (deforestation, illegal mining, loss of land, etc.) inspired the first author in advancing biodiversity research. UITOTO can be downloaded from its GitHub repository (at https://github.com/atorresgalvis/UITOTO) or directly installed by entering the following commands into the R console:
   install.packages("devtools")

   library(devtools)

   devtools::install_github("atorresgalvis/UITOTO")




**Fig. 1:**
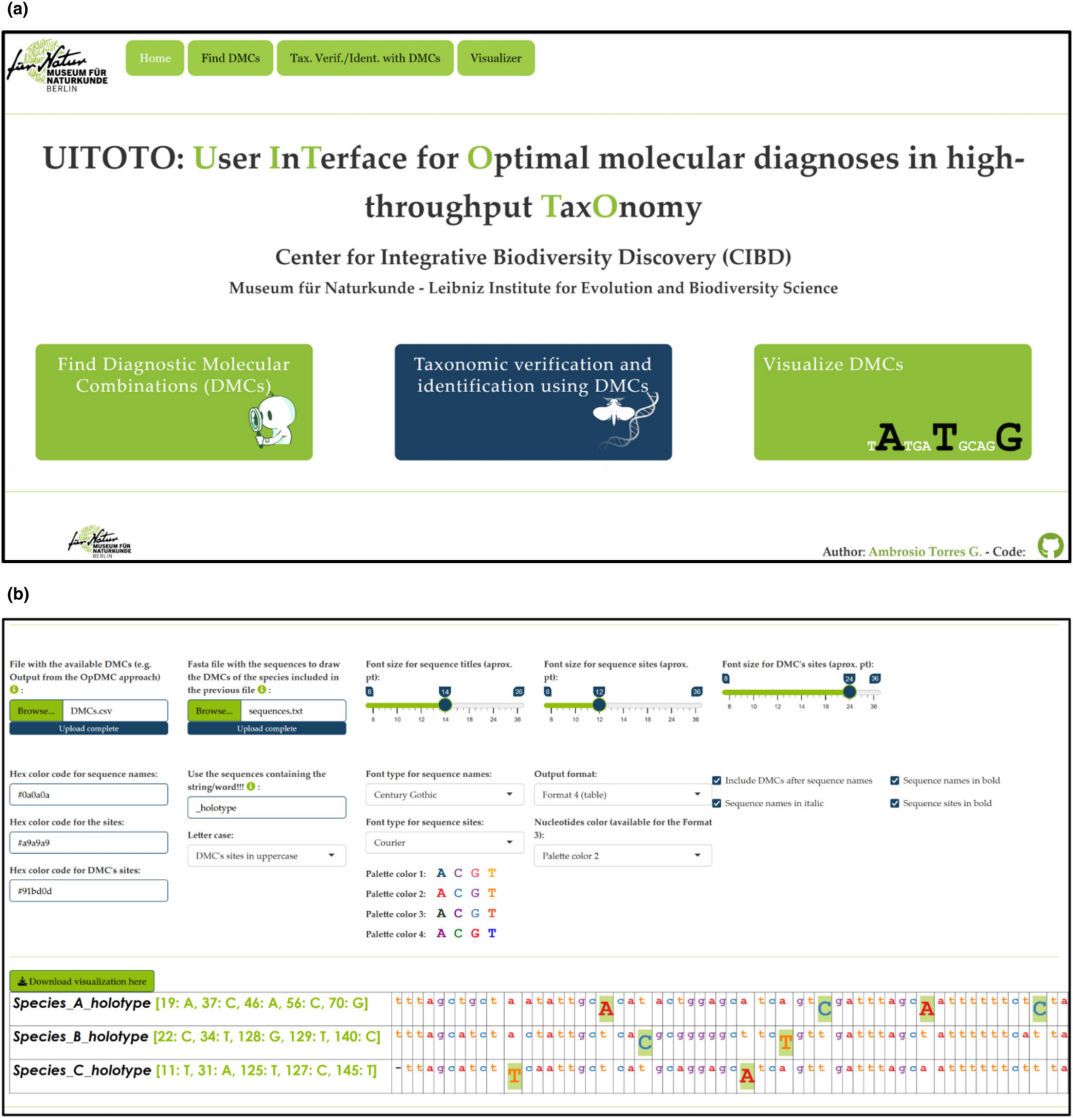
(a) UITOTO Shiny app home page. Available at https://atorresgalvis.shinyapps.io/MolecularDiagnoses/. (b) Module “Visualization of DMCs” of the Shiny app. See also the GitHub repository of UITOTO at https://github.com/atorresgalvis/UITOTO.

**Fig. 2:**
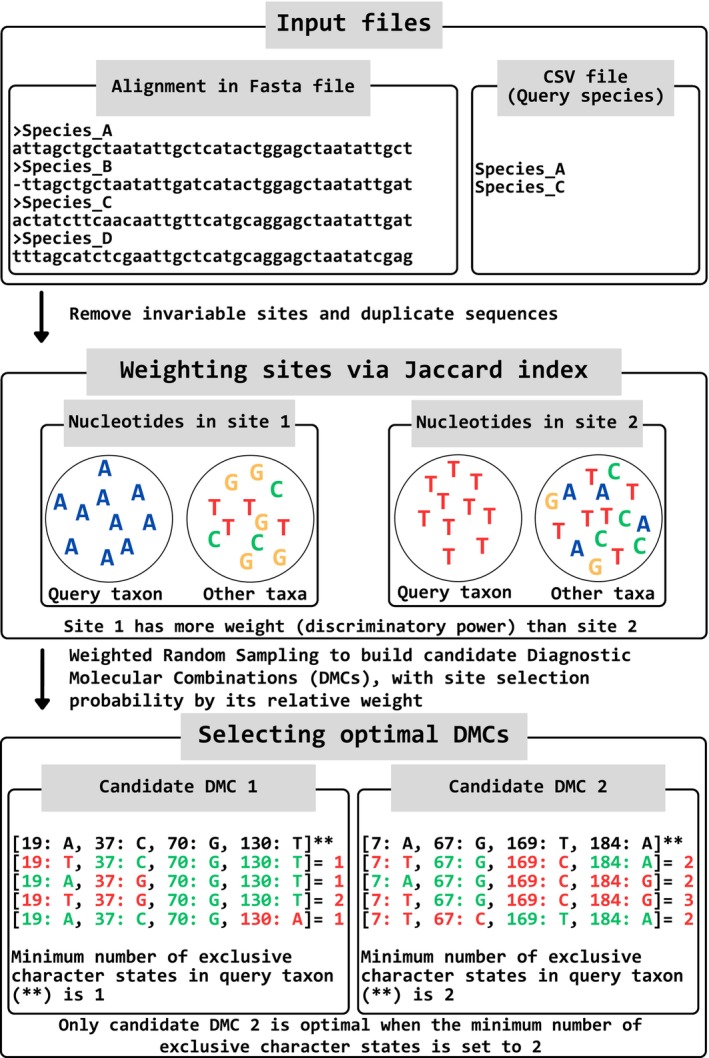
The fundamental algorithmic process utilized by UITOTO to identify optimal Diagnostic Molecular Combinations (DMCs). The queries represent the entities to be diagnosed, such as new species to be described.

The software requires the pre‐installation of some R packages such as Biostrings (Pagès et al., 2024), DECIPHER (Wright, 2016), dplyr (Wickham et al., 2023), ggplot2 (Wickham, 2016), readr (Wickham et al., 2024), SeqinR (Charif and Lobry, 2007), shiny (Chang et al., 2024), shinyjs (Attali, 2021), and shinyWidgets (Perrier et al., 2024). Therefore, we recommend following the instructions of the UITOTO repository in GitHub. UITOTO also features a user‐friendly Shiny app (Chang et al., 2024) accessible online (Fig. 1. https://atorresgalvis.shinyapps.io/MolecularDiagnoses/) or locally in RStudio (Posit team, 2023). The UITOTO repository in GitHub also includes example datasets for testing the software, along with a user manual that introduces the three main modules. Note that all input files should be properly formatted. For example, the module for the “Identification of Diagnostic Molecular Combinations” (DMCs; see below) requires two files: (i) an alignment in FASTA format containing the query and reference sequences, and (ii) a CSV file listing the query taxa for which DMCs should be identified. The query list can include species names (e.g. *Diachlorus_curvipes*) if DMCs are to be found at the species level, or higher‐level taxa such as genera (e.g. *Diachlorus*) if broader diagnostic markers are required (see Fig. 2, and the example files of the GitHub repository).

### Module 1: Identification of diagnostic molecular combinations (DMCs)

This is the core module of UITOTO and is responsible for finding optimal DMCs. DMC searches start by activating “Find Diagnostic Molecular Combinations (DMCs)” in the UITOTO Shiny app. Alternatively, users can also use the OpDMC function included in the package. This method assigns a weight to each site based on the Jaccard index of similarity, which measures the number of sequences that differ from the query taxon (e.g. sequences of a new taxon) for the nucleotide at this site (Jaccard, 1908). The function then employs a Weighted Random Sampling approach to construct candidate combinations for DMCs. Sites with higher uniqueness values are then more likely to be included in the candidate DMCs (Fig. 2). Fedosov *et al*.'s MOLD (Fedosov et al., 2022) also uses a Jaccard index of similarity to measure the uniqueness of the sites (in their terms, “score”). However, it does not use a Weighted Random Sampling approach for building the candidate combinations. Instead, “The scores are then ranked in descending order and the user defines how many of the top‐ranking sites are used for assembling a draft combination” (Fedosov et al., 2022). This approach could lead to problems when dealing with multiple ties, as several sites could have the same weights (or scores); that is, some sites with high uniqueness values might not be sampled at all if they fall below the user‐defined threshold. Therefore, UITOTO probabilistically samples from all sites rather than relying on a strict cut‐off and is thus less likely to overlook informative sites, especially when few sites have good Jaccard Index values. UITOTO thus evaluates a larger number of different candidate DMCs.

Before the DMCs search begins, UITOTO asks the user to specify the minimum number of exclusive character states (i.e. nucleotides) desired for candidate DMCs. For example, specifying a minimum of two exclusive character states means that an optimal DMC must differ by at least two sites from the sequences of all other species (Fig. 2). This criterion ensures significant differentiation from other taxa while using a higher number may invalidate a DMC when additional intraspecific sequence variability is found in the future. This approach is analogous to the Bremer support measure in phylogenetic inference, which quantifies the number of extra steps required to break a tree node (Bremer, 1994). For the purpose of molecular diagnoses, UITOTO applies a similar principle by considering the number of extra nucleotide matches needed to break the specificity of a DMC (Fig. 2).

Next, the software identifies those sites that are most frequently included in candidate DMCs that meet the preceding criterion of having a minimum number of exclusive character states. These sites are then used to build one final majority‐consensus DMC for each species, which is then confirmed to still meet the user‐specified exclusivity criterion. As output, UITOTO generates a CSV file containing the final identified DMCs. In addition, it also provides one of the optimal combinations as an alternative DMC for each species. Users can adjust various settings to control the DMC search process, including the number of candidate DMCs to test (i.e. iterations), the minimum length for the DMCs and the minimum number of exclusive character states of the DMC (see the instructions in the GitHub repository and the package manual).

### Module 2: Verification of DMCs and their use for specimen identification

The verification tool allows the user to test DMCs against user‐supplied data from private or public databases. This tool is also accessible via both the Shiny app's graphical interface (under the module “Taxonomic verification and identification using DMCs for aligned and unaligned sequences”) and the command‐line version, which integrates three different functions included in the package: ALnID, Identifier and IdentifierU.

The Identifier function compares DMCs against a set of sequences and identifies those that match the DMCs. It generates a detailed output file that highlights which sequence is a match. In contrast, the ALnID function employs dynamic programming (a variation of the Needleman and Wunsch, 1970 algorithm for global alignment) from the R package DECIPHER (Wright, 2016) to align unaligned sequences from a FASTA file with the sequences used to derive the DMCs. It then operates similarly to the previously described Identifier function. Lastly, the IdentifierU function employs an alignment‐free approach, using a dynamic sliding window to iteratively compare DMCs with different parts of individual sequences. This function allows users to set a mismatch threshold and thus enhance flexibility in taxonomic identification and verification (see the available instructions in the GitHub repository and the package manual).

This comprehensive verification and identification framework is unique to UITOTO and critical for assessing the reliability of molecular diagnoses, because rigorous cross‐validation requires software capable of analysing hundreds of thousands of aligned and unaligned sequences. By comparing identified specimens with known taxonomic assignments, users can efficiently evaluate the accuracy and robustness of each DMC and refine their selection criteria across datasets and taxonomic groups. Other software packages such as BLOG (Weitschek et al., 2013) and CAOS‐R (Sarkar et al., 2008; Bergmann, 2024) also offer specimen identification, but they do not use DMCs (see Table 1).

### Module 3: Visualization of DMCs

The “Visualization of DMCs” module generates customizable, publication‐quality comparisons through interactive features that are accessed via the graphical user interface of the UITOTO Shiny app. It improves how users interpret and communicate DMCs that are distributed across long DNA sequences (Fig. 1b). Comparable visualization functionality is currently available only in UITOTO and DeSignate (Hütter et al., 2020) (see Table 1).

### Performance test of UITOTO and MOLD

#### Test datasets

• As the first test dataset, we used a published DNA barcode library that covers 97% of the 459 species of European Lepidoptera (hereafter, *European Butterflies*; Dincă et al., 2021). We extracted the data for the 49 butterfly species occurring in the United Kingdom (UK) and then derived the DMCs based on only the 591 COI sequences from the UK (hereafter, “European butterflies training dataset”). To assess the performance of the DMCs based on UK data, we then used all European data (“European butterflies testing dataset”) comprising 21 483 unaligned sequences of the butterfly species presented in the rest of Europe. This also included additional sequences for the 49 species occurring in the UK. The barcode lengths ranged from 113 to 1498 base pairs (bp) (mean = 656 bp).
• The second dataset pertains to a real dataset used for a monographic treatment of Mycetophilidae (Diptera: Bibionomorpha) from Singapore (Amorim et al., 2025). To identify the DMCs, we used 1456 aligned COI minibarcodes for 118 species (313 bp: called the “Mycetophilidae training dataset”). The DMCs were then compared against 60 349 unaligned Mycetophilidae sequences from The Barcode of Life Data System (BOLD; Ratnasingham and Hebert, 2007: “Mycetophilidae testing dataset”). The length of the sequences in the latter ranged from 498 to 658 base pairs (mean = 605).
• The third dataset was used to delimit 69 new species of the genus *Megaselia* (Diptera: Phoridae) from Sentosa Island, Singapore (Lee, 2023). DMCs are here proposed based on 2229 aligned COI sequences (hereafter, the “Megaselia training dataset”). To evaluate the reliability of the DMCs for the 69 species, we employed an additional dataset of 30 289 aligned Phoridae sequences from other parts of Singapore and Indonesia (hereafter, the “Megaselia testing dataset”). Both datasets consisted of minibarcodes with a length of 313 base pairs.

The selected datasets enabled us to assess UITOTO across different taxonomic contexts and analytical challenges. The European butterfly dataset is taxonomically nearly complete and supported by a well‐curated barcode library. Deriving DMCs from UK species and testing them against the full European dataset simulates expanded sampling that increases both intraspecific variability, which can reduce conspecific identification accuracy, and interspecific diversity. This makes it more difficult to find species‐specific DMCs. In contrast, the Mycetophilidae and *Megaselia* datasets represent large‐scale projects on dark taxa with incompletely known faunas, allowing us to evaluate UITOTO in rapidly growing barcode libraries typical for newly described or poorly known groups. Finally, the tests include both aligned and unaligned sequences to mirror the heterogeneity of publicly available barcode data, which often differ in length and may require complex alignment procedures.

#### Obtaining Diagnostic Molecular Combinations (DMCs)

DMCs obtained with UITOTO were compared with DMCs obtained with MOLD (Fedosov et al., 2022) and/or BLOG (Weitschek et al., 2013). We only tested BLOG for one dataset, because the software was not included in Fedosov et al. (2022) study, which proposed MOLD and compared it against other available software and demonstrated that MOLD outperformed DeSignate (Hütter et al., 2020) for medium‐ and high‐complexity datasets. We here only assessed BLOG for the *Megaselia* dataset to establish whether it outperforms MOLD. We used the default settings of MOLD and BLOG (available at https://github.com/SashaFedosov/MolD/blob/master/MolD_parameters.txt and http://dmb.iasi.cnr.it/blog.php) and found that MOLD greatly outperformed BLOG (see Table S1, Supplementary Material online), so that we dropped BLOG from further comparison with UITOTO. We also did not include other tools such as CAOS‐R (Sarkar et al., 2008; Bergmann, 2024), QUIDDICH (Kühn and Haase, 2020), FASTACHAR (Merckelbach and Borges, 2020) and Spider (Brown et al., 2012), as these software packages can only identify single nucleotide diagnostic characters. However, the use of single character‐DMCs in a diagnosis is generally discouraged because it renders diagnoses very sensitive to sampling.

To compare the DMCs obtained with MOLD and UITOTO we varied the UITOTO search settings in the OpDMC function (see above and the package manual). All searches treated gaps as missing data and employed 50 000 iterations (number of candidate DMCs to evaluate) and a refinement strength of 0.33. The refinement strength, which ranges from 0 to 1, controls the proportion of sub‐combinations from each optimal DMC that is tested. The higher the refinement strength, the greater the likelihood of identifying shorter DMCs (though this also increases the computational time). Each run had its own set of defined parameters to control the minimum length of the DMCs (**MnLen**) and their minimum number of exclusive character states (**exclusive**. See Fig. 2). The basic command used can be summarized as follows:
   OpDMC("Alignment.fasta", "Queries.csv", iter = 50000, **MnLen** = 2, **exclusive** = 2, RefStrength = 0.33, OutName = "OutputFile.csv", GapsNew = FALSE)




We conducted seven searches: The minimum length of the DMCs was fixed to six (i.e. **MnLen** = 6) for three searches, but the number of exclusive character states varied between 2 and 4 (i.e. **exclusive** = 2, 3 and 4, respectively). For the remaining three searches, we retained the same number of exclusive character state settings (i.e. **exclusive** = 2, 3 and 4, respectively) but allowed the **MnLen** to vary freely; that is, the minimum possible length of a DMC obtained with **exclusive** = 2 was also two. When the user sets an **exclusive** value higher than the **MnLen** value, the function automatically adjusts **MnLen** to match the **exclusive** value, thus also allowing the minimum length to vary freely. Finally, we conducted one search with a minimum length of ten (i.e. **MnLen** = 10) and set the number of exclusive character states to four (i.e. **exclusive** = 4).

The Supplementary Material contains the scripts we used in this study. It also provides detailed instructions for identifying the DMCs in UITOTO (ScriptSearchDMC.R). For comparison with MOLD, we used the default settings of this software, which are identical to those used in Fedosov et al. (2022: section 2.3.1). MOLD allows for adjusting additional parameters; when it comes to DMC length, the software only allows setting a maximum length while it does not permit specifying a minimum length or a minimum number of exclusive character states. In contrast, UITOTO allows fine‐tuning both parameters, providing greater flexibility in optimizing DMCs. This is a major advantage because our results demonstrate that MOLD tends to produce short DMCs (see Discussion and Conclusions section below) lacking specificity. Consequently, even if MOLD's available settings were adjusted, this would not lead to a significant improvement in DMC quality, as its optimization criteria inherently favour shorter DMCs.

#### Testing the reliability of DMCs

To test the reliability of the DMCs in our study, we used two UITOTO functions depending on whether the test sequences were aligned (Identifier) or unaligned (ALnID). The Identifier function was applied to the *Megaselia* test dataset while the two other datasets were analysed with AnID, because they included unaligned sequences. The latter function first aligns the sequences of the testing dataset with those used to derive the DMCs (i.e. those from the training dataset) and then operates similarly to the Identifier function. In all cases, we conducted the cross‐validation analyses using two different tolerances: one allowing up to one mismatch between the DMCs and the sequences and the other with zero tolerance for mismatches (see the script ScriptIdentification.R in the Supplementary Material online). Note that for clarity and conciseness, we present only the results allowing for up to one mismatch, as the zero‐tolerance scenario produced qualitatively the same patterns across all three datasets examined (see Table S2, Supplementary Material online).

**Fig. 3:**
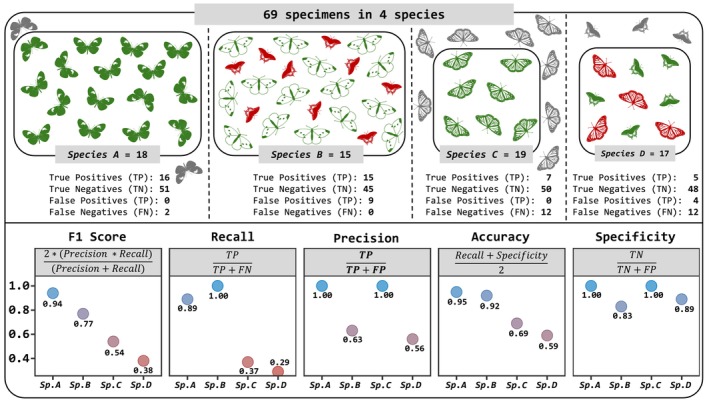
Comparison of the performance of commonly used metrics (F1 Score, Recall, Precision, Accuracy and Specificity) in classification techniques, applied to a hypothetical dataset of 69 specimens across four species. Green silhouettes: correctly identified specimens; red silhouettes: misidentified specimens; grey silhouettes: unclassified specimens. Insect silhouette images sourced from https://www.phylopic.org/.

To summarize the results, we developed the R function ConfuClass.R (Supplementary Material). It processes the output files from the ALnID and Identifier functions to generate confusion matrices and classification metrics, including precision, recall, specificity, accuracy, and F1 score (see below for the equations). The ConfuClass function detects when sequences to be identified contain missing data at DMCs sites and excludes them from the calculations. These metrics, commonly used in machine learning (e.g. discriminant analysis) and deep learning, provide insights into the effectiveness of classification models (Fig. 3). “Recall” refers to the proportion of correctly identified specimens out of all the specimens that belong to a particular species. For example, a recall of 50% indicates that the DMC was only able to assign a species label to half of the test specimens from the species. “Precision” indicates the proportion of specimens that were correctly identified (i.e. long diagnoses tend to suffer from low recall but have high precision). “Specificity” refers to the proportion of specimens correctly identified as not belonging to a particular species, that is, it indicates how well a DMC can avoid false positives for a given species. DMCs with high recall often have low specificity because they are so general that they assign the same species label to multiple species. Finally, the F1 score combines precision and recall into a single metric. In our case, the metrics are calculated for each DMC corresponding to an individual species, ensuring a tailored assessment of the performance for each species (see the file ScriptConfuClassi.R in the Supplementary Material online). Note that if no conspecific sequences were present in the testing datasets, only “Specificity” was used to evaluate the DMC, as the number of true positives and false negatives could not be determined.

In evaluating the performance of the software packages, we focus on **F1** and **Accuracy** as they summarize the performance values obtained across all metrics. However, detailed results for all metrics are available in the Tables S2 and S3. Overall, we would argue that the F1 Score is preferable over accuracy for datasets, especially when a few species are very common and have a lot of sequences while most are only represented by 1–2 barcodes. The F1 Score is less sensitive to such imbalances (i.e. a high probability of having many more true negatives than true positives). This is shown in Fig. 3. The DMC for species A performs well, but this is only expressed by the F1 score, while accuracy for species A and B is very similar although the DMC for species B leads to 9 false positives.

• Recall = True Positives/(True Positives + False Negatives)
• Precision = True Positives/(True Positives + False Positives)
• Specificity = True Negatives/(True Negatives + False Positives)
• Accuracy = (Recall + Specificity)/2
• F1 Score = 2 * (Precision * Recall)/(Precision + Recall)

## Results

**Fig. 4:**
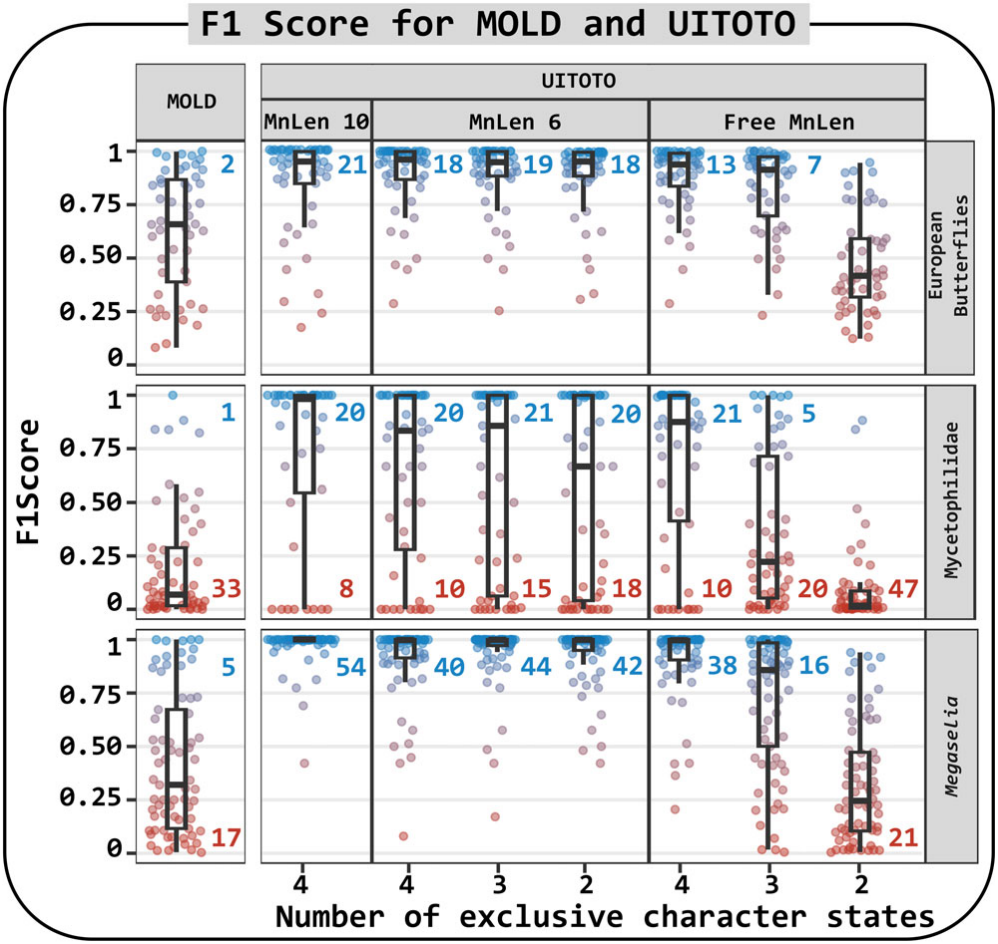
F1 Score of the diagnostic molecular combinations (DMCs) from MOLD and UITOTO for the three datasets. Each point represents a query species. Due to overlap, the blue numbers indicate the number of species with values greater than (>) 0.99, while the red numbers indicate those with values less than (<) 0.10. The number of exclusive character states and MnLen (minimum length of the DMCs) are settings available in UITOTO (i.e. not applicable for MOLD)

The F1 Score results across the European butterflies, Mycetophilidae and *Megaselia* datasets revealed two patterns (Fig. 4): (i) longer diagnostic molecular combinations (DMCs) consistently produced higher F1 Scores across all analyses and (ii) DMCs generated by UITOTO were generally more reliable than those obtained from MOLD (F1 Score average = 0.20–0.63). Specifically, we observed that F1 Scores increased with DMC lengths in UITOTO. The highest F1 Scores were obtained by setting a fixed Minimum Length (MnLen) of DMCs to 10 nucleotides (on average 0.72–0.97), followed by a MnLen of six nucleotides (on average 0.56–0.94), with the lowest F1 Scores resulting when we allowed the MnLen to vary freely (on average 0.09–0.92). Additionally, we observed a positive correlation between the number of exclusive character states and F1, a pattern particularly evident when we allowed the MnLen to vary freely (Fig. 4; see also Tables S2 and S3, Supplementary Material online).

The use of Accuracy as evaluation criterion yielded somewhat different results (Appendix S1, Supplementary Material online). As shown in Fig. 3 (compare species A and B), Accuracy carries a low penalty for generating false positives (assigning the wrong specimens to a species). Not surprisingly, short DMCs thus tend to have higher Accuracy values without being more accurate in a common‐sense interpretation of the word. The generally short DMCs of MOLD nevertheless did not consistently outperform the generally longer DMCs generated by UITOTO (see Tables S2 and S3, Supplementary Material online). Instead, the Accuracy values of MOLD DMCs (on average 0.956–0.992) were comparable to or even lower than those obtained with UITOTO when the MnLen was allowed to vary freely (0.897–0.996) or set to short lengths by specifying a low number of exclusive character states (e.g. 0.880–0.988 with exclusive = 2). This is particularly pronounced for two of the three datasets; *viz*. European butterflies and *Megaselia* (Table S3, Supplementary Material online).

Regarding the remaining metrics, we observe that short DMCs have high Recall, while longer DMCs tend to yield higher Precision and Specificity (Appendix S2 and S4, Supplementary Material online. See also Fig. 3). Overall, the performance of the DMCs is highly dataset specific. For example, the results for the Mycetophilidae dataset were the least satisfying (Fig. 4; Appendices [Link], [Link] and Tables S2 and S3, Supplementary Material online). We suspect that this is due to high intraspecific sequence variability in many species (Amorim et al., 2025; Meier et al., 2025). In deriving DMCs, it is always important to consider the specific characteristics and scope of each dataset. Intra‐ and interspecific sampling differ and should be considered when defining search parameters.

## Discussion and Conclusions

Our empirical tests based on three datasets with different properties reveal that UITOTO produces more reliable Diagnostic Molecular Combinations (DMCs) than currently available software (e.g. MOLD: Fedosov et al., 2022; BLOG: Weitschek et al., 2013). This consistent result is particularly relevant given that our three empirical datasets represented different realistic challenges. The European butterfly dataset had dense coverage of species and intraspecific variability and was thus best suited to test whether molecular diagnoses are feasible for a taxonomically very well‐known taxon. In contrast, the *Megaselia* (Phoridae) and the mycetophilid datasets represent poorly known “dark taxa” with more than 90% of the species‐level diversity being undescribed. The *Megaselia* dataset pertains to a taxon with low intraspecific sequence variability, while the Mycetophilidae dataset is characterized by high intraspecific variability (Amorim et al., 2025; Meier et al., 2025). Yet, UITOTO outperformed MOLD for all three datasets because it tends to generate short DMCs. This increases the chance for assigning a sequence to a query species but also leads to a significant number of false positives (i.e. erroneous species identifications or incorrect taxonomic assignments. See Fig. 3). For example, the DMCs obtained by MOLD for *Megaselia* ranged in length from two to six sites (average = 3.5). In contrast, UITOTO's DMCs were longer (3–8 sites: average = 4.6), when requiring two exclusive states per species and increased to 5–11 sites (average = 4.6) when requiring four exclusive states.

This preference for shorter DMCs leads to considerably lower F1 Scores for DMCs obtained with MOLD, which seems to struggle with balancing precision, specificity, and recall (see Fig. 3). MOLD's performance could theoretically be improved by incorporating a parameter to control the minimum DMC length. However, MOLD would still lack the option to define the number of exclusive character states in each species‐specific DMC. This feature directly assesses the DMC performance while MOLD uses simulations to generate sequences with introduced mutations to test the robustness of the DMCs. In addition, the ability to set the number of exclusive sites indirectly controls DMC length (e.g. a DMC with four exclusive character states cannot have fewer than four sites). Such dual control over specificity and length helps with the generation of good DMC candidates by reducing the likelihood of false positives and false negatives. Additional unique features of UITOTO are the comprehensive DMC verification module and the visualization module for obtaining publication‐ready diagnoses.

**Fig. 5:**
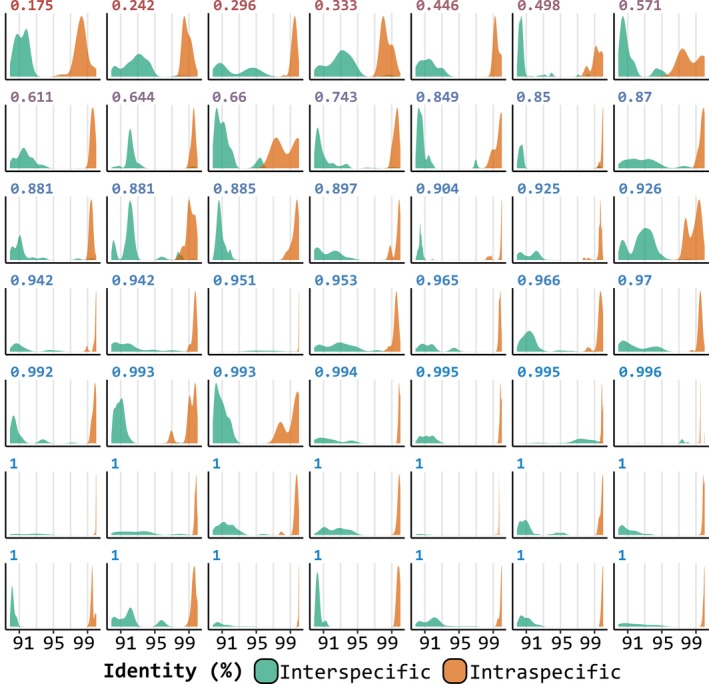
Comparison of classification results from diagnostic molecular combinations (DMCs) and the Basic Local Alignment Search Tool (BLAST. Camacho et al., 2009) in the European butterfly dataset. The performance of the DMCs was evaluated using the F1 Score, while BLAST performance was assessed by the percentage of identical nucleotides between two sequences (identity percentage).

Overall, UITOTO has so many features because it addresses the challenge of managing the trade‐off between overly detailed diagnoses that may exclude some specimens and overly general ones that might include non‐members (see Figs 3 and 4). The software is built around the principle that users must explore multiple parameter combinations during DMC searches to identify settings that yield robust diagnoses for their dataset. As a starting point, we recommend using four exclusive character states and 10 nucleotides as the minimum DMC length, because this combination frequently produces the best‐performing DMCs. Afterwards, the users can systematically relax or tighten these criteria to better accommodate genetic variation. The need for such flexibility becomes apparent in taxa such as Mycetophilidae, for which optimal DMCs (i.e. high F1 Scores) were difficult to obtain (Fig. 4), presumably because delimited species exhibit high intraspecific variability (Amorim et al., 2025). Taxonomists will be familiar with the analogous situation in morphology: diagnoses must accommodate different levels of intraspecific variation in different groups. Some “recalcitrant” species have high intraspecific variability and low differentiation from close relatives. In such cases, UITOTO's capacity to adjust settings is essential, as illustrated by Amorim et al. (2025), who achieved optimal DMCs for newly described Mycetophilidae species using tailored parameter combinations (mean F1 Score = 0.92; mean accuracy = 0.99). Finally, our analyses indicate that even high‐quality DMCs should be complemented with diagnostic information from other character systems. This is mandatory for species that share DNA barcodes (Meier, 2008), but is probably also necessary for species with high intraspecific COI variability and low interspecific differentiation. One should not expect that all species have satisfying DMCs based on a partial COI sequence. To rigorously derive DMCs, we therefore introduced a cross‐validation module. It reveals that certain species belonging to the *Megaselia* and European Butterfly datasets yielded DMCs with low F1 Scores (e.g. <0.75). A detailed analysis using the Basic Local Alignment Search Tool (BLAST; Camacho et al., 2009) revealed that this was particularly common for those species with high intraspecific variability and small barcode gaps that were here defined as the difference between the distributions of inter‐ and intraspecific genetic identities or distances (see Fig. 5). Interestingly, we also identified several species, where BLAST failed to yield identification at a reasonable identity threshold (e.g. 99%) while DMCs were able to assign unidentified sequences correctly to species (see Fig. 5). This highlights that the currently well entrenched habit of only identifying specimens with BLAST should be complemented with specimen identification based on DMCs.

Overall, our tests also reiterate the importance of sampling. Reliable DMCs will be elusive when the sequence diversity in a training dataset is artificially low due to insufficient intra‐ and interspecific sampling. This is analogous to morphological diagnoses whose accuracy is heavily dependent on sampling. This is one important reason why DMCs need to be tested against a large number of sequences from query and non‐query taxa (as suggested by Weitschek et al., 2013). Dense taxon sampling helps to mitigate the risk of obtaining DMCs that yield false positives because too few sequences were used for generating the diagnoses.

Most of the issues discussed here will be familiar to taxonomists who prepare morphological diagnoses. There is no fixed standard for how many characters are required, and taxonomists intuitively adjust the number of exclusive states depending on context: species in species‐rich genera often require longer diagnoses, whereas members of morphologically distinct or monotypic genera may need only a few. The same principle applies to DNA barcodes, but the balancing act can be more quantitative because DMCs can be evaluated systematically across settings using performance metrics such as accuracy and F1 Score. Multiple combinations of minimum length and minimum exclusive character states can be tested to identify the configurations that yield the highest‐performing DMCs. F1 Scores can then be used to eliminate DMCs with high false‐positive rates, which inflate the workloads of taxonomists by requiring the reidentification of many misassigned specimens. Arguably, false negatives are more desirable because they are likely to represent “unusual” specimens.

Finally, while UITOTO was originally developed with a focus on animals, its modular design and underlying principles are equally suitable for fungal and plant taxonomy, including the automation of approaches such as those proposed by Tedersoo et al. (2024) and Flores‐Olvera et al. (2016). UITOTO brings together functions for identifying, testing, and visualizing DMCs in a single framework, which makes it a practical platform for taxonomists who need to evaluate molecular diagnoses rigorously. The Shiny app interface further expands accessibility by allowing users to run analyses and generate publication‐quality visualizations without requiring command‐line experience. We hope that this flexibility will encourage adoption by a wide range of users and support the application of molecular diagnoses across diverse empirical and theoretical datasets.

## Supporting information


**Table S1.** Diagnostic molecular combinations (DMCs) for the Megaselia dataset derived by BLOG.

**Table S2.** Classification statistics of the diagnostic molecular combinations (DMCs) for the three datasets using zero tolerance for mismatches.

**Table S3.** Classification statistics of the diagnostic molecular combinations (DMCs) for the three datasets using one (1) site of tolerance for mismatches.

**Appendix S1.** Accuracy of the Diagnostic Molecular Combinations (DMCs) from MOLD and UITOTO for the three datasets. Each point represents a query species. Due to overlap, the blue numbers indicate the number of species with values greater than (>) 0.99, while the red numbers indicate those with values less than (<) 0.10. The number of exclusive character states and MnLen (minimum length of the DMCs) are settings available in UITOTO (i.e. not applicable for MOLD).

**Appendix S2.** Recall of the Diagnostic Molecular Combinations (DMCs) from MOLD and UITOTO for the three datasets. Each point represents a query species. Due to overlap, the blue numbers indicate the number of species with values greater than (>) 0.99, while the red numbers indicate those with values less than (<) 0.10. The number of exclusive character states and MnLen (minimum length of the DMCs) are settings available in UITOTO (i.e. not applicable for MOLD).

**Appendix S3.** Precision of the diagnostic molecular combinations (DMCs) from MOLD and UITOTO for the three datasets. Each point represents a query species. Due to overlap, the blue numbers indicate the number of species with values greater than (>) 0.99, while the red numbers indicate those with values less than (<) 0.10. The number of exclusive character states and MnLen (minimum length of the DMCs) are settings available in UITOTO (i.e. not applicable for MOLD).

**Appendix S4.** Specificity of the diagnostic molecular combinations (DMCs) from MOLD and UITOTO for the three datasets. Each point represents a query species. Due to overlap, the blue numbers indicate the number of species with values greater than (>) 0.99, while the red numbers indicate those with values less than (<) 0.10. The number of exclusive character states and MnLen (minimum length of the DMCs) are settings available in UITOTO (i.e. not applicable for MOLD).

**Data S1.** Scripts.

## Data Availability

The data that support the findings of this study are available in the supplementary material of this article.
