# Supplementary figures and images for: UITOTO: a software for generating molecular diagnoses for species descriptions
Source: Cladistics. 2025 Dec 20;42(2):193–205. doi: 10.1111/cla.70023 (PMC12977935; doi:10.1111/cla.70023)

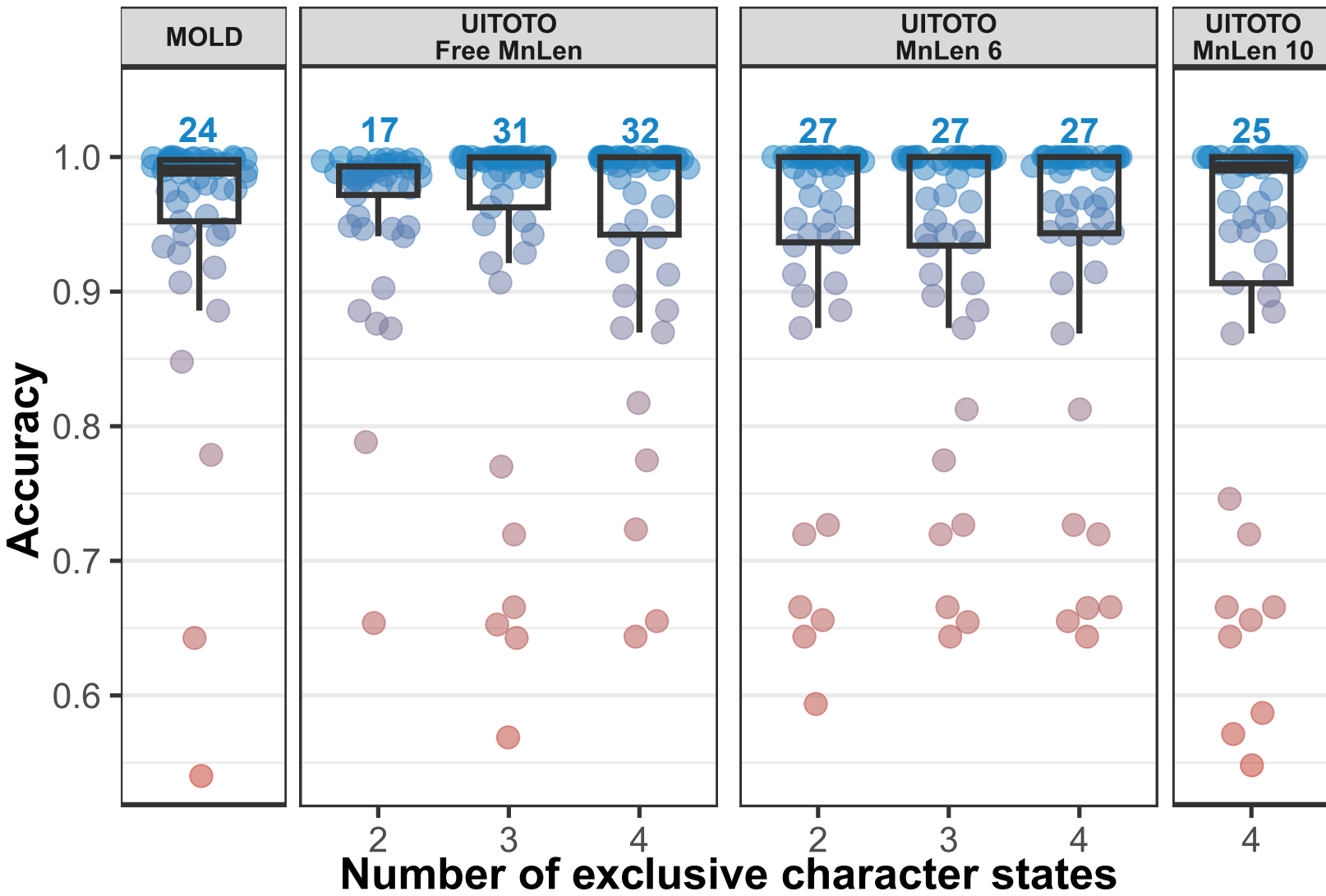

Accuracy

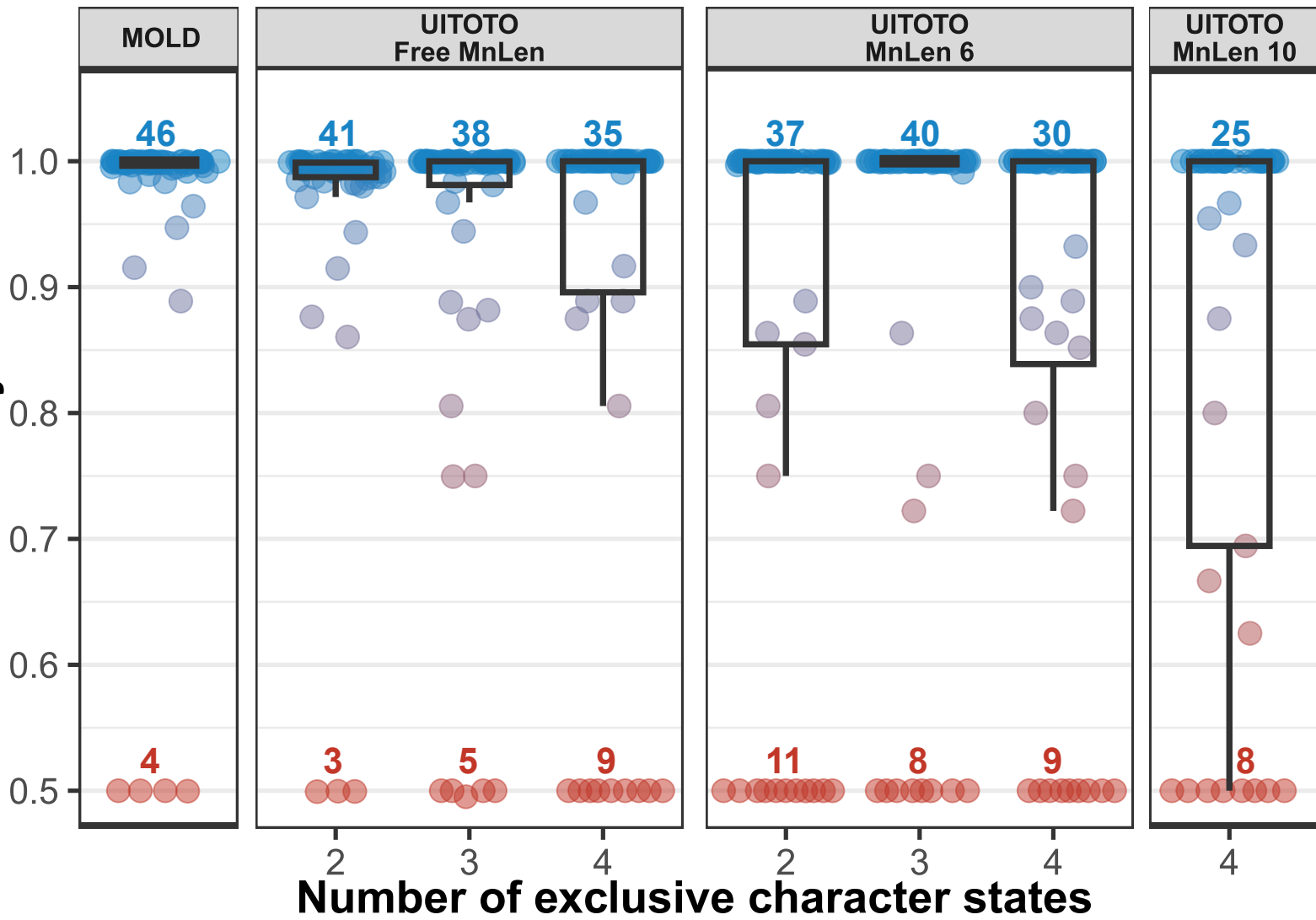

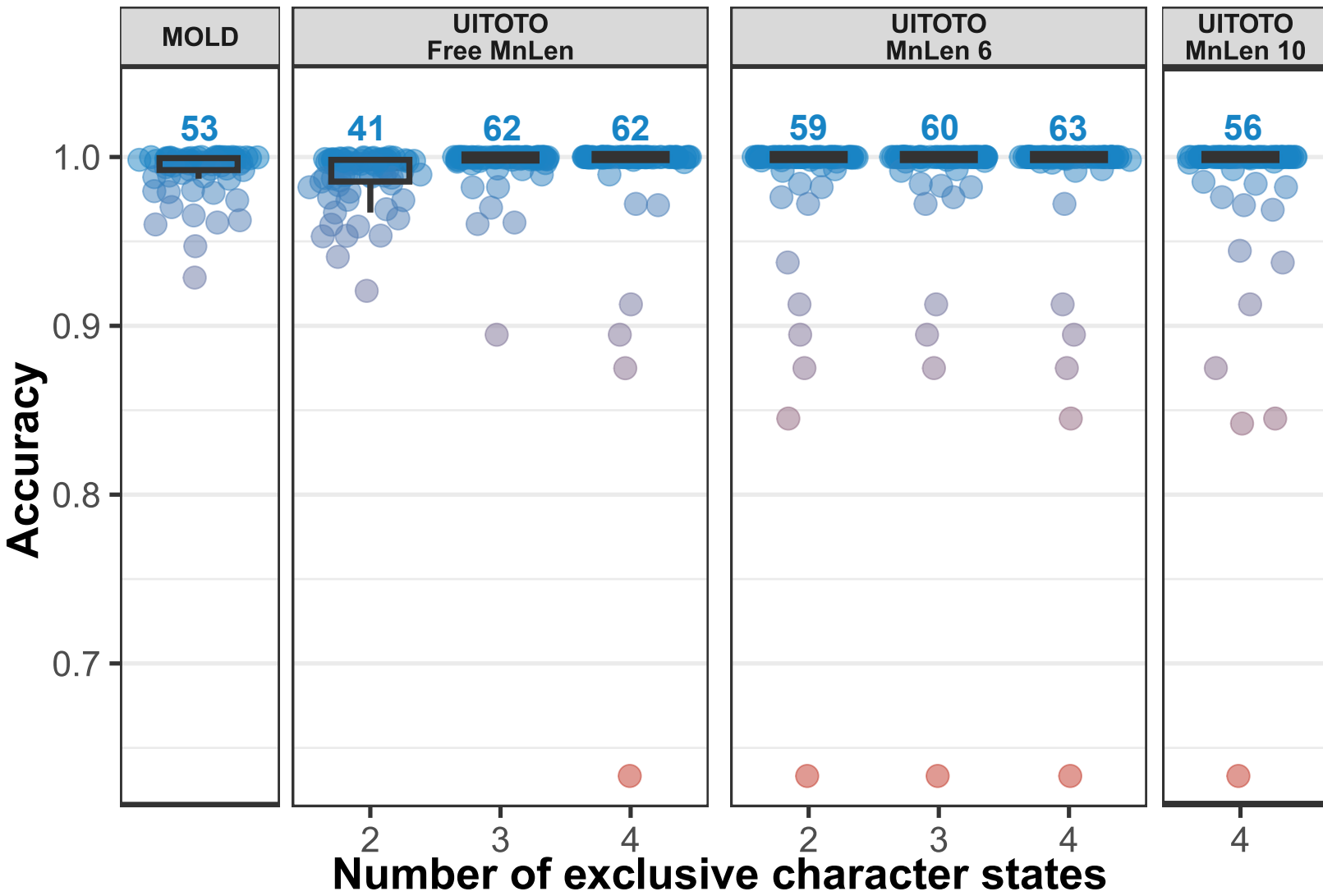

Supplement: Supplementary file 4 — Appendix S1. Accuracy of the Diagnostic Molecular Combinations (DMCs) from MOLD and UITOTO for the three datasets. Each point represents a query species. Due to overlap, the blue numbers indicate the number of species with values greater than (>) 0.99, while the red numbers indicate those with values less than (<) 0.10. The number of exclusive character states and MnLen (minimum length of the DMCs) are settings available in UITOTO (i.e. not applicable for MOLD). [file CLA-42-193-s007.pdf]

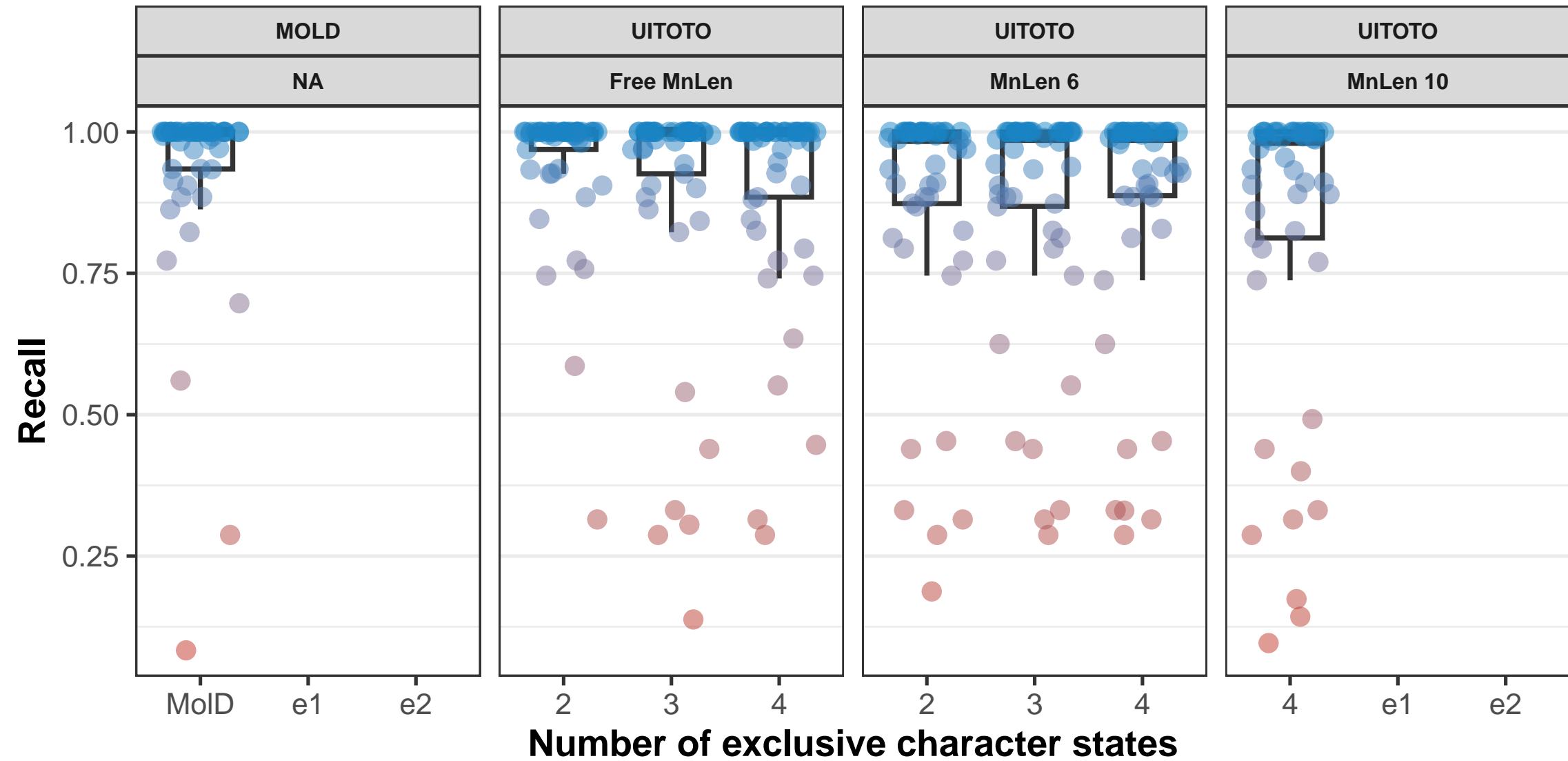

Recall

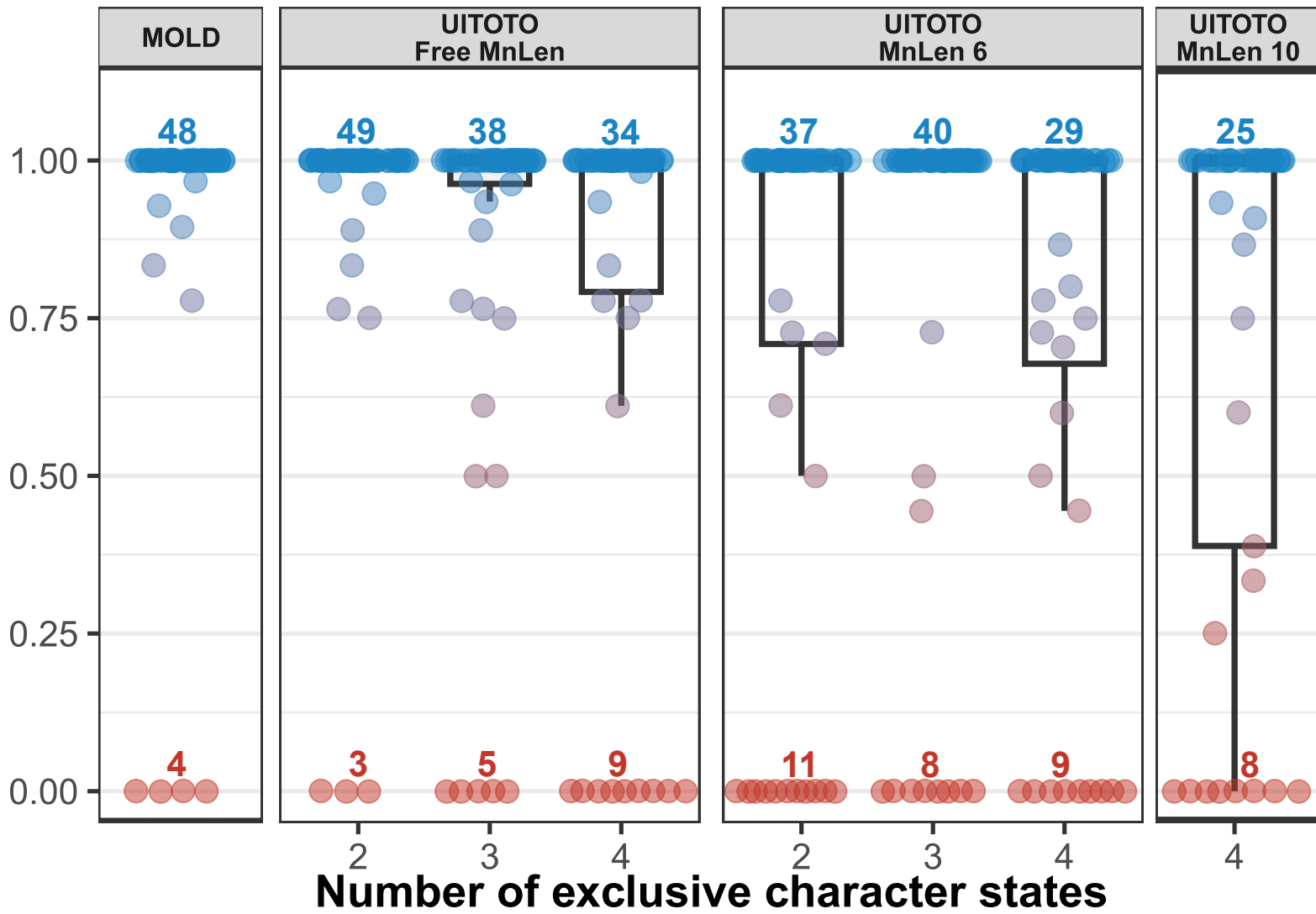

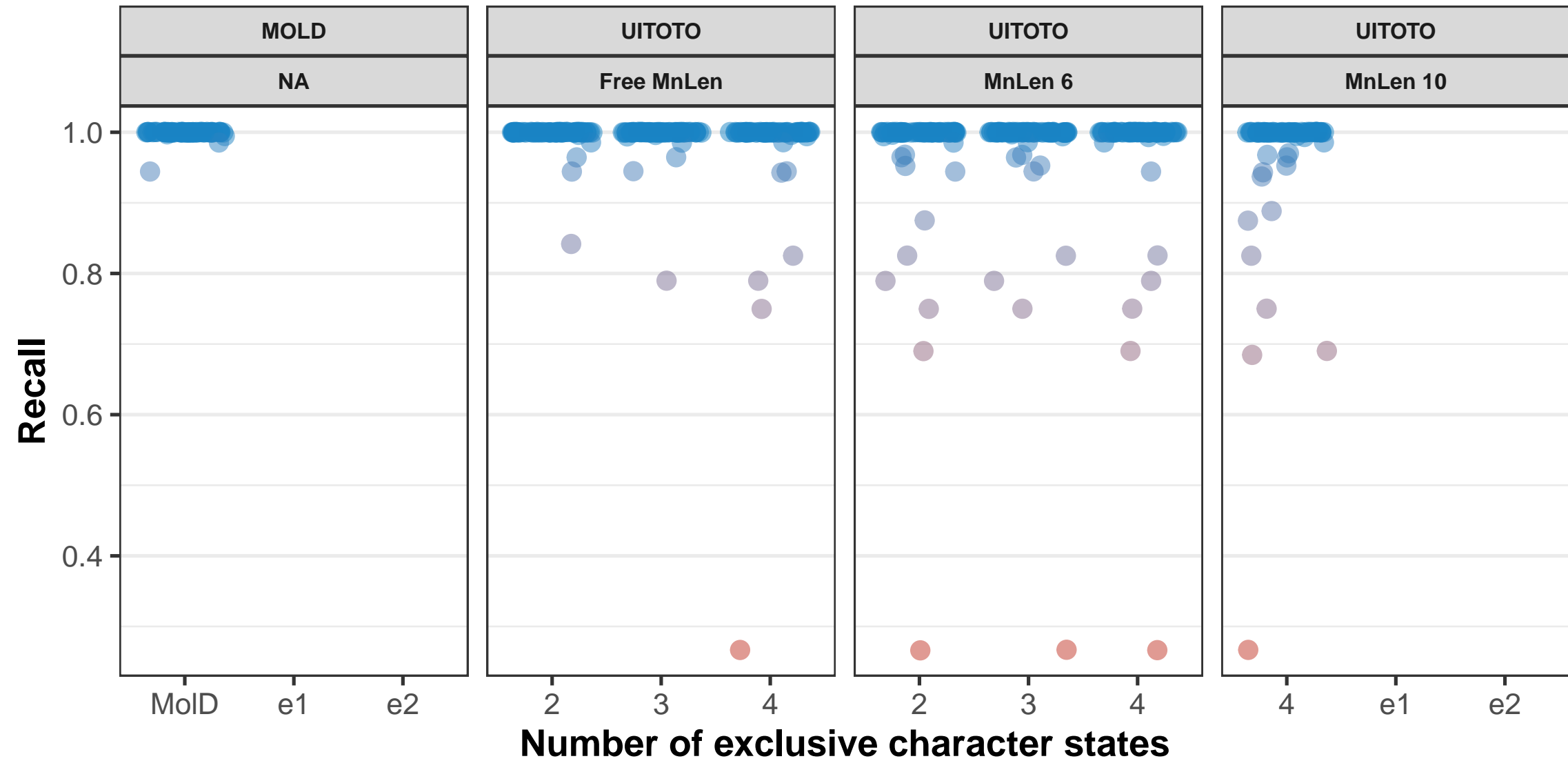

Supplement: Supplementary file 5 — Appendix S2. Recall of the Diagnostic Molecular Combinations (DMCs) from MOLD and UITOTO for the three datasets. Each point represents a query species. Due to overlap, the blue numbers indicate the number of species with values greater than (>) 0.99, while the red numbers indicate those with values less than (<) 0.10. The number of exclusive character states and MnLen (minimum length of the DMCs) are settings available in UITOTO (i.e. not applicable for MOLD). [file CLA-42-193-s003.pdf]

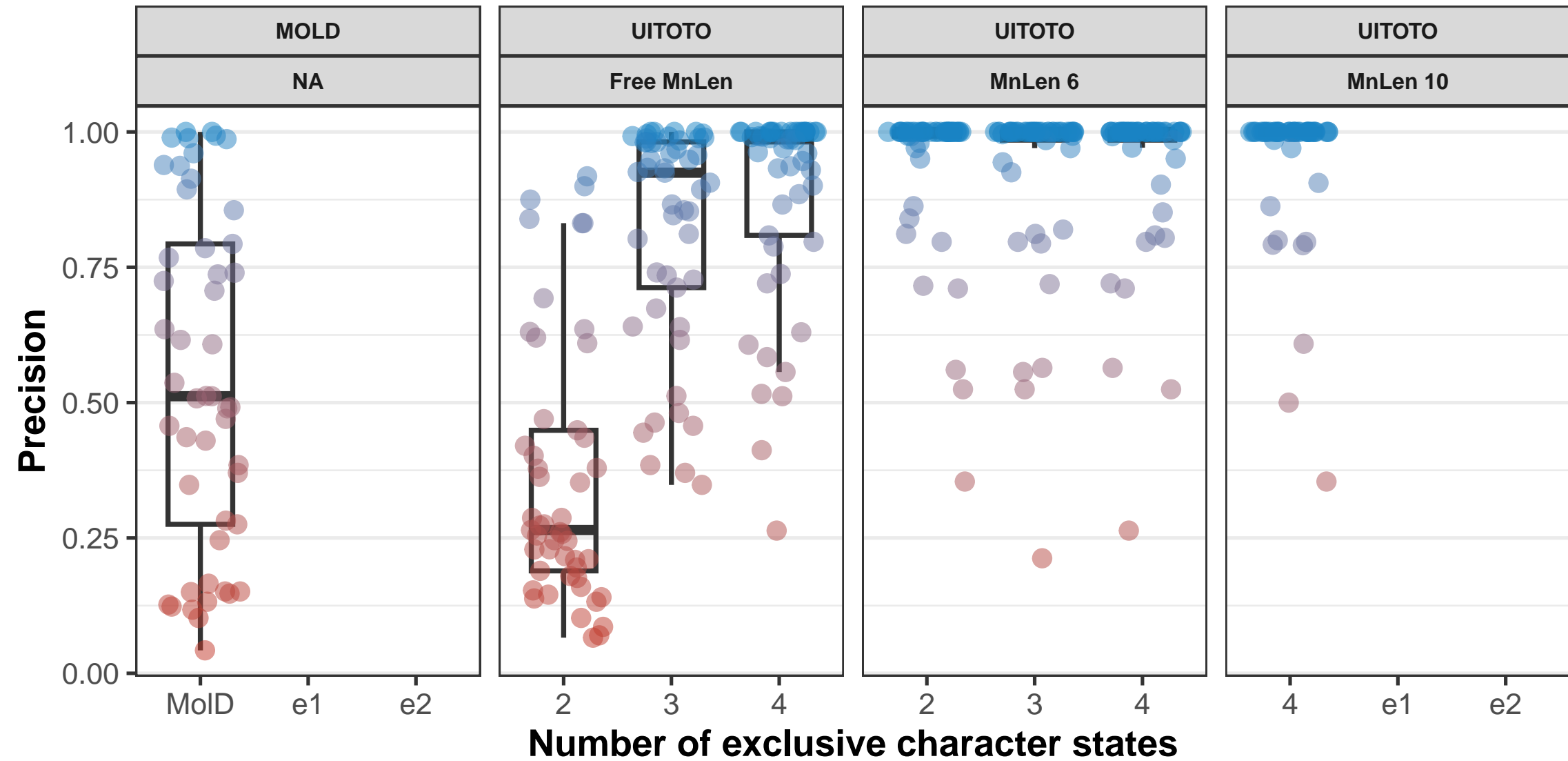

Precision

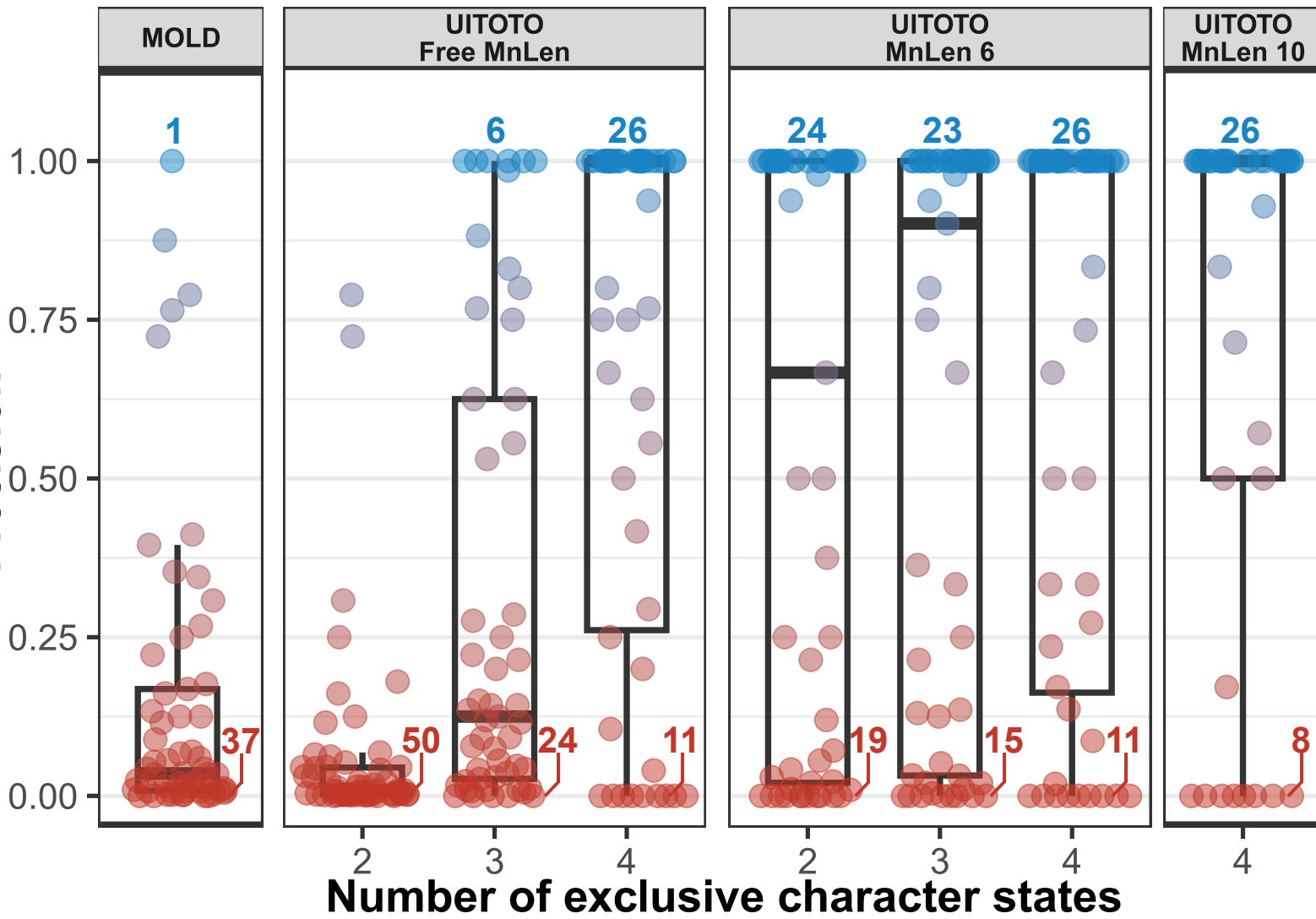

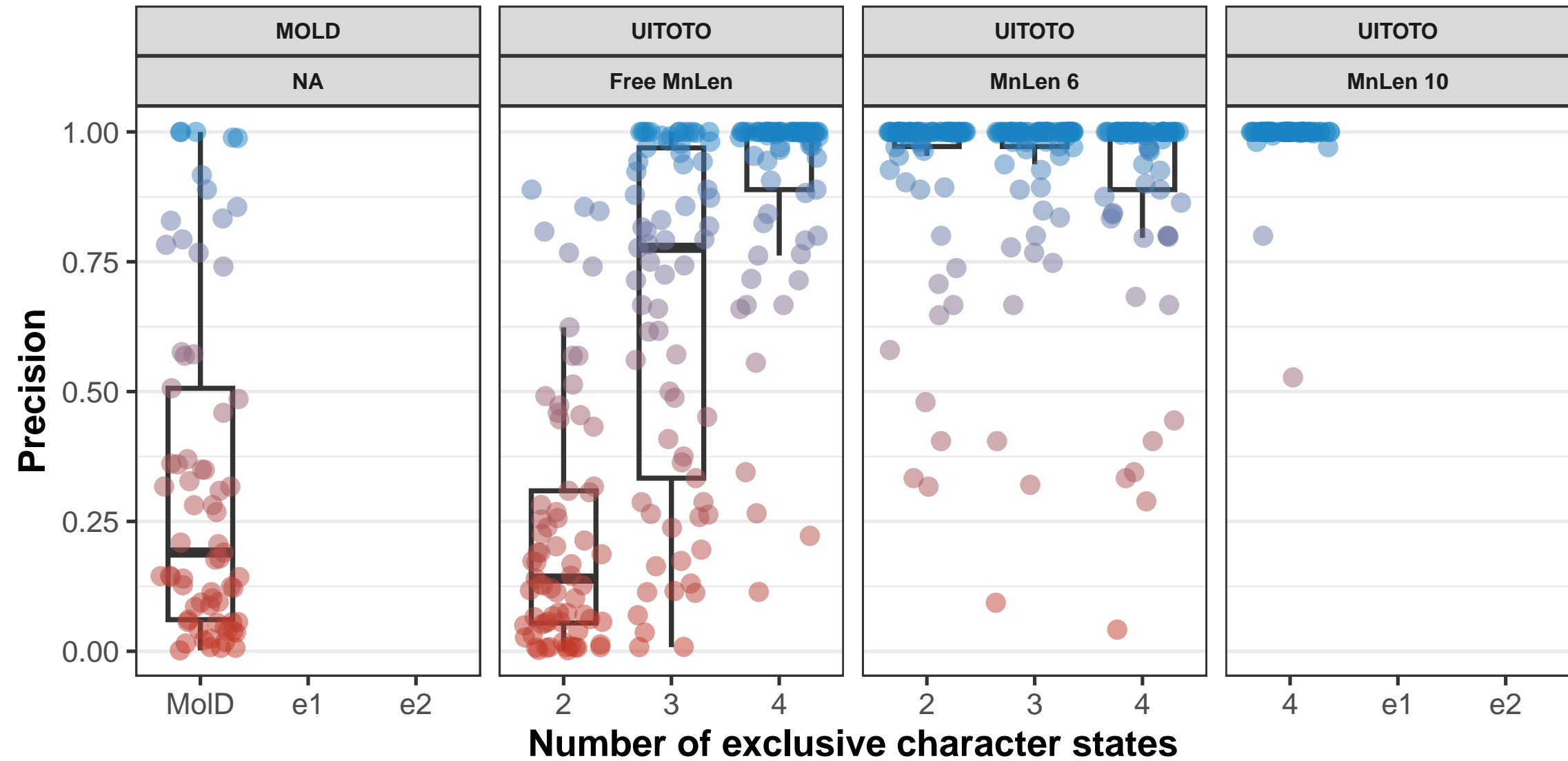

Supplement: Supplementary file 6 — Appendix S3. Precision of the diagnostic molecular combinations (DMCs) from MOLD and UITOTO for the three datasets. Each point represents a query species. Due to overlap, the blue numbers indicate the number of species with values greater than (>) 0.99, while the red numbers indicate those with values less than (<) 0.10. The number of exclusive character states and MnLen (minimum length of the DMCs) are settings available in UITOTO (i.e. not applicable for MOLD). [file CLA-42-193-s002.pdf]

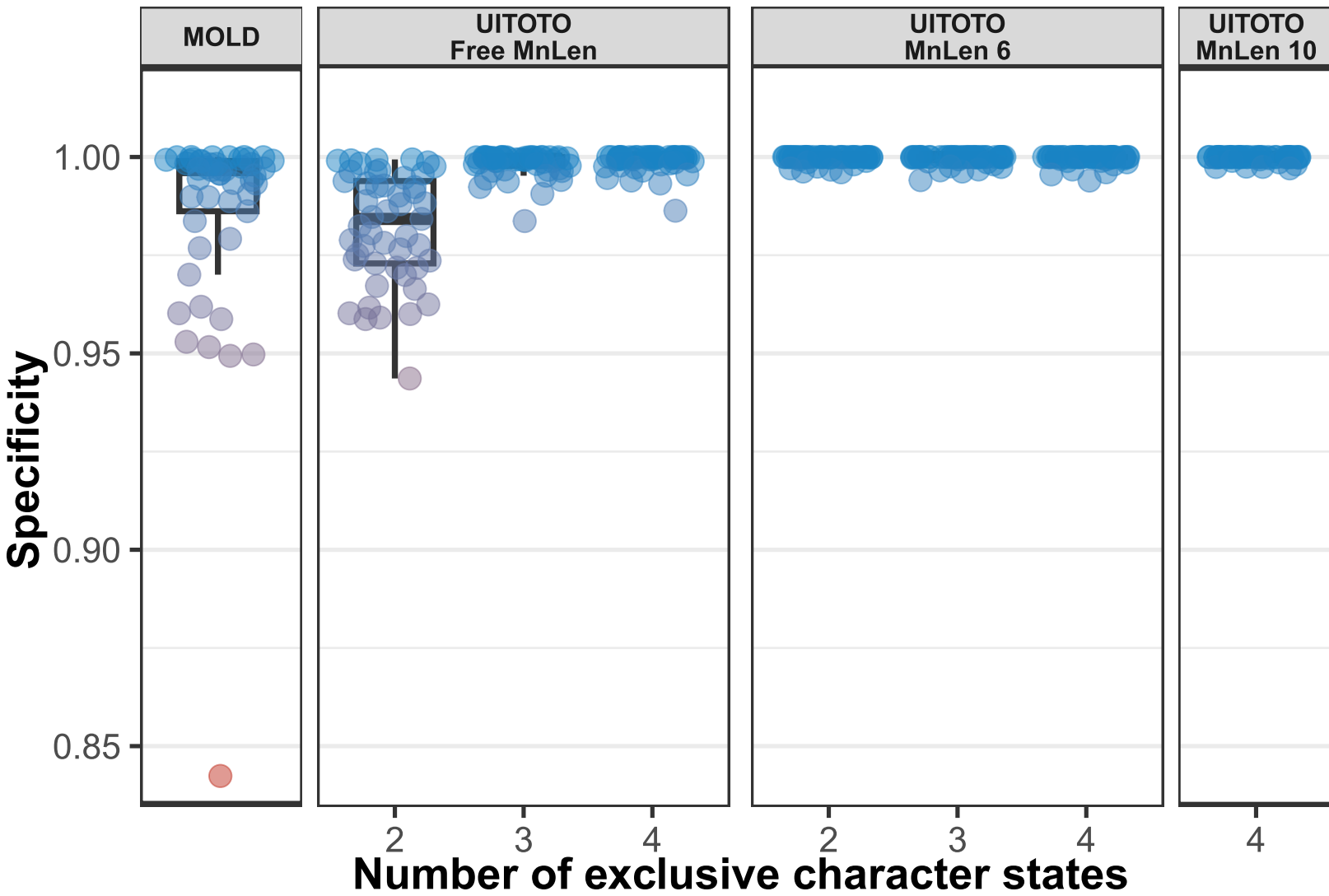

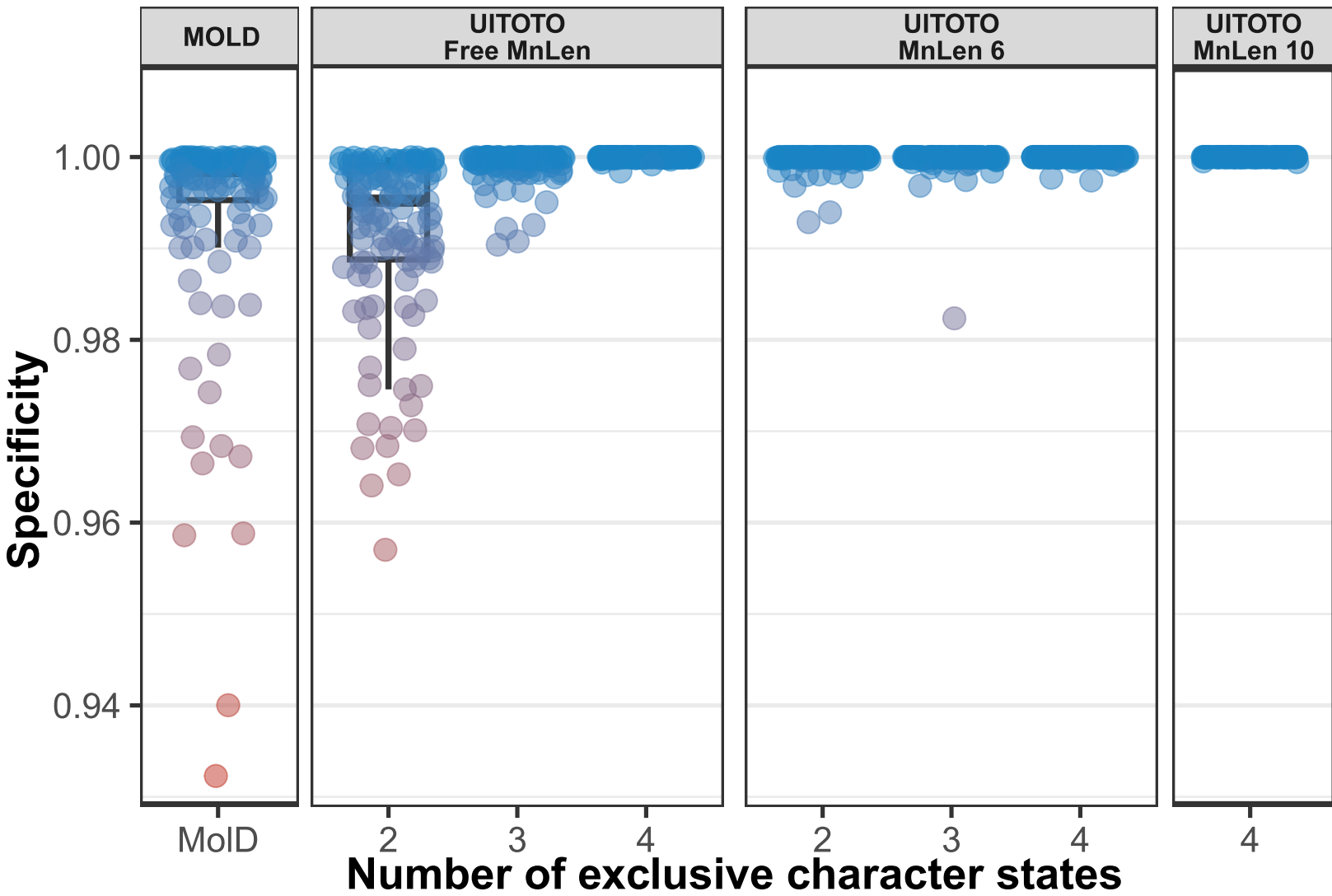

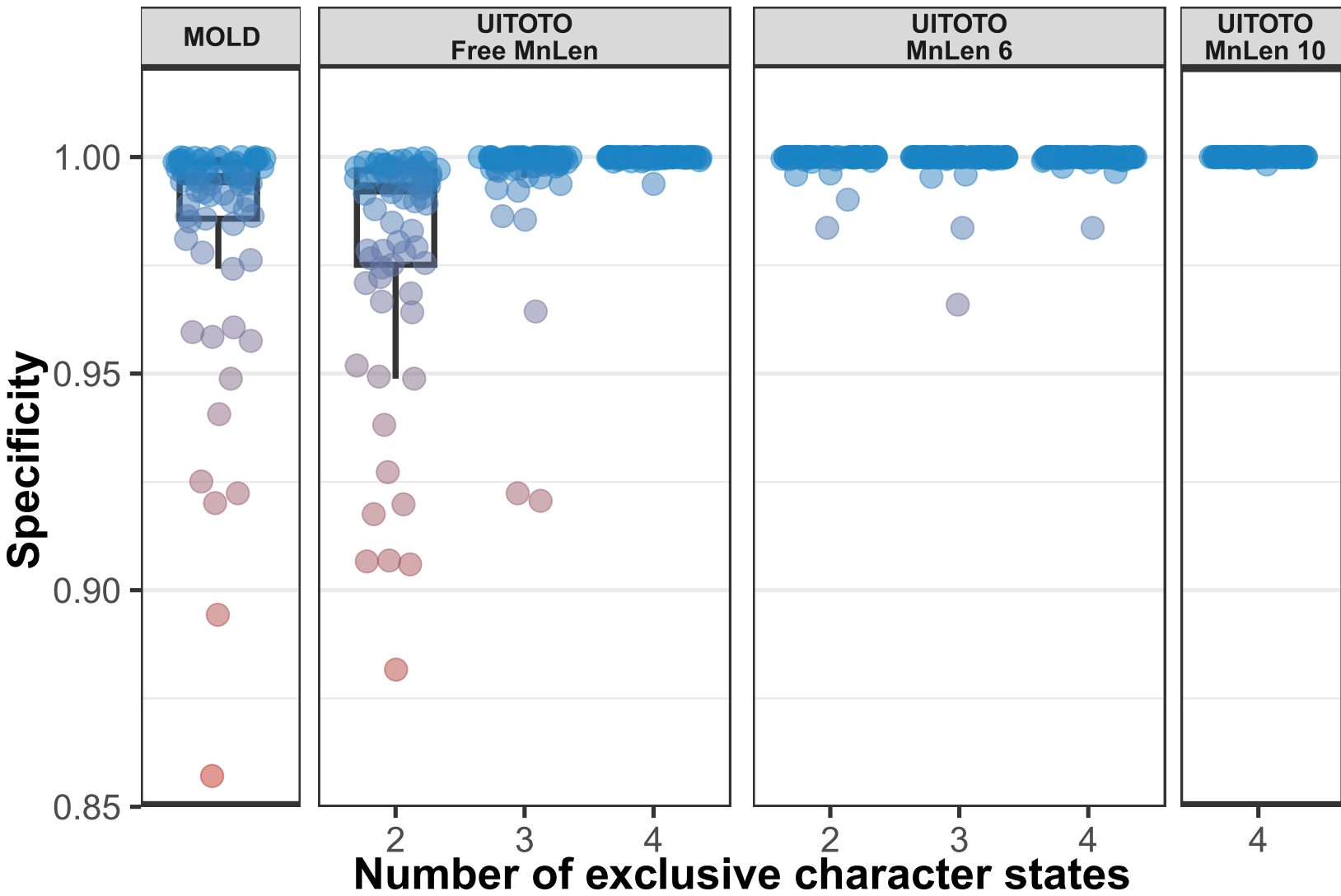

Supplement: Supplementary file 7 — Appendix S4. Specificity of the diagnostic molecular combinations (DMCs) from MOLD and UITOTO for the three datasets. Each point represents a query species. Due to overlap, the blue numbers indicate the number of species with values greater than (>) 0.99, while the red numbers indicate those with values less than (<) 0.10. The number of exclusive character states and MnLen (minimum length of the DMCs) are settings available in UITOTO (i.e. not applicable for MOLD). [file CLA-42-193-s004.pdf]
